# Detection of the *DGAT1* Gene Variation and Its Association with Growth Traits in Degeres Sheep

**DOI:** 10.3390/ani16162583

**Published:** 2026-08-19

**Authors:** Ainagul Begenova, Kaster Nurgulsim, Gulbadan Otepova, Aiganym Bekenova, Roman Bissengaliyev, Zhassulan Baibolin, Talgat Kulmagambetov

**Affiliations:** Institute of Animal Science and Veterinary, Saken Seifullin Kazakh Agrotechnical University Astana 010011, Kazakhstan; arsen_arlan@mail.ru (G.O.); aiganym5555@mail.ru (A.B.); bissengaliyev.r@gmail.com (R.B.); zhasulan.baibolin@gmail.com (Z.B.); t.kulmagambetov@kazatu.edu.kz (T.K.)

**Keywords:** Degeres sheep, MAS, SNP, association, growth traits

## Abstract

In Kazakhstan, meat–fat-tailed sheep breeding is crucial for breed development and high-quality mutton production. Understanding variations in candidate genes may improve selection for meat quality and growth traits. Therefore, this study aimed to investigate the relative mRNA expression levels of the *DGAT1*, *CAST*, *IGF-1*, *POU1F1* genes using qRT-PCR in tissues from three 12-month-old male Degeres sheep, identify SNP variations by PCR and Sanger sequencing, and assess their associations with growth traits in 18-month-old female Degeres sheep (*n* = 380). Relative mRNA expression revealed tissue-specific expression patterns of the studied genes, with *DGAT1* showing the highest expression in abdominal fat (*p* < 0.001), *CAST* in the abomasum (*p* < 0.01), *IGF-1* in the liver (*p* < 0.001), and *POU1F1* in the testis (*p* < 0.05). The findings uncovered a previously reported synonymous SNP (c.1461 C > T) in the *DGAT1* gene and polymorphism information content (PIC) exhibited low polymorphism. Moreover, the identified SNP deviated from Hardy–Weinberg equilibrium (HWE) and was significantly associated with chest width (*p* = 0.001), with the “TT” genotype exhibiting greater chest width. Therefore, further studies with larger sample sizes are required to validate its potential use in marker-assisted selection (MAS) to optimize sheep breeding.

## Simple Summary

Sheep are an important source of meat in Kazakhstan, and improving their growth and meat quality can benefit both farmers and consumers. One way to achieve this is by identifying genes that influence these traits. In this study, we examined four genes involved in growth, muscle development, and fat metabolism in Degeres sheep. We measured how these genes were expressed in different body tissues and searched for polymorphisms that are associated with growth traits. We identified a genetic variation in one gene (*DGAT1*) that was associated with chest width, an important growth characteristic. The results suggest that this variant might be useful for selecting sheep with desirable growth traits in future breeding programs. Additional studies involving larger numbers of animals are needed to confirm these findings before they can be applied in sheep breeding.

## 1. Introduction

The advancement of meat–fat-tailed sheep breeding plays a vital role in livestock production, providing an important foundation for developing new breeds and ensuring a stable supply of high-quality, affordable mutton in Kazakhstan. The Degeres sheep are a semi-fine wool breed developed in Kazakhstan between the 1930s and 1980s through crossbreeding Shropshire and Precoce sheep with Kazakh fat-tailed ewes. In terms of wool quality and productivity, it is considered one of the leading fat-tailed sheep breeds in the world [1]. Therefore, elucidating the genetic potential of this breed is essential, as it may serve as a valuable genetic resource for the genetic improvement of other sheep populations.

Marker-assisted selection (MAS) allows breeders to identify and select animals with superior genetic traits at an early age, thereby accelerating genetic improvement compared to traditional selection methods [2]. In this case, studying genetic polymorphism, particularly single-nucleotide polymorphisms (SNPs), in genes associated with production traits in sheep is crucial. Identifying SNPs in genes associated with production traits also provides candidate loci for future validation and breeding applications [3,4]. Nevertheless, additional functional studies and validation in independent populations are required before statistically associated SNPs can be considered causal variants or applied in breeding programs.

Although numerous candidate genes have been associated with growth and carcass traits in sheep, *DGAT1*, *CAST*, *IGF1*, and *POU1F1* were selected because of their well-established roles in lipid metabolism, muscle development, endocrine regulation, and growth signaling. Studying previously reported SNP variants within these genes allows us to evaluate their effects on growth, carcass, and production traits in a different genetic background with distinct allele frequencies and selection history.

Diacylglycerol O-acyltransferase 1 (DGAT1) catalyzes the final step in triglyceride synthesis, playing a critical role in fat metabolism [5]. It was reported that *DGAT1* locus is related to tail length [6] and fat-tail weight in sheep [7]. Also, *DGAT1* was linked to milk production traits in other livestock [8].

Furthermore, polymorphisms in the calpastatin (*CAST*) gene have been associated with variations in meat quality and tenderness [9]. *CAST* encodes an inhibitor of calpain, a key proteolytic enzyme involved in postmortem muscle protein degradation, which plays a central role in determining meat tenderness and muscle development [10].

Insulin-like growth factor 1 (IGF1) plays a role in growth promotion, insulin signaling, and development [11]. SNPs in *IGF1* were associated with growth rates and carcass traits in various sheep [12,13]. In addition, the relation of *IGF1* to meat or carcass traits has been extensively studied in beef cattle and chickens [14,15].

Pituitary-specific transcription factor 1 (POU1F1) regulates the expression of growth hormone, prolactin, and other key pituitary hormones. It is well-known that genetic variations in this gene cause hypopituitarism in human [16]. In sheep, polymorphism within the *POU1F1* gene showed significant effect on weaning weight [17]. In goat, it was identified as candidate gene for carcass, growth traits and litter size [18,19].

Gene expression analysis provides insight into the transcriptional activity of candidate genes and their potential roles in biological processes underlying growth traits, whereas SNP association analysis identifies genetic variants associated with phenotypic variation. Combining these approaches enables the evaluation of both functional gene expression and genetic polymorphisms, providing complementary evidence for the involvement of candidate genes in growth performance. Therefore, the aim of this study was to analyze the relative mRNA expression levels of the *DGAT1*, *CAST*, *IGF1*, and *POU1F1* genes, identify SNPs in these genes, and assess their associations with growth traits in Degeres sheep. The findings of this study may contribute to identifying traits that can be more effectively improved through molecular breeding strategies.

## 2. Materials and Methods

### 2.1. Sample Preparation and Data Collection

All experimental procedures involving animals were reviewed and approved by the Local Ethics Committee on Biological and Medical Ethics of Saken Seifullin Kazakh Agrotechnical University (Protocol No. 1, dated 16 October 2024). Blood samples were collected from all healthy, 18-month-old female Degeres sheep (*n* = 380) maintained at the “Qazaq Steppe Sheep” farm under standard management conditions and raised on pasture. All animals meeting the predefined inclusion criteria (breed, sex, age, and health status) were included in the study. Body measurement traits, including body weight (BW), height at the withers (HW), chest circumference (ChC), diagonal body length (DBL), chest depth (ChD), chest width (ChW), cannon circumference (CC) were measured in accordance with established standards.

In addition, after slaughtering of 12-month-old male Degeres sheep (*n* = 3), samples were collected from the muscle, heart, kidney, lung, testis, subcutaneous fat, abdominal fat, liver, small intestine, and abomasum. The tissue samples were promptly frozen in liquid nitrogen and stored at −80 °C until further use.

### 2.2. RNA Extraction and qRT-PCR

Tissue samples of Degeres sheep were obtained post-slaughter. Total RNA was extracted using RecoverAll™ Total Nucleic Acid Isolation Kit for FFPE (Thermo Fisher Scientific, Waltham, MA, USA; REF: AM1975) according to the manufacturer’s protocol for non-formalin-fixed tissues, with the deparaffinization steps omitted. RNA concentration was measured by NanoDrop™ 1000 spectrophotometer (Thermo Scientific, Waltham, MA, USA) and its integrity was assessed by electrophoresis on 1.0% agarose gel. Complementary DNA (cDNA) was synthesized from the extracted RNA using RevertAid First Strand cDNA Synthesis Kit (Thermo Fisher Scientific, Waltham, MA, USA; REF: K1621). PCR amplification efficiency was evaluated by standard curve analysis using serial dilutions of cDNA on the QuantStudio™ Real-Time PCR System (Applied Biosystems, Thermo Fisher Scientific, Waltham, MA, USA). Melt curve analysis was performed following amplification to confirm a single specific amplicon.

Based on the mRNA sequences of the *ovine DGAT1* (XM_060419894.1), *CAST* (NM_001009788.1), *IGF-1* (XM_060411609.1), *POU1F1* (NM_001009350.2) genes, primers for real-time quantitative polymerase chain reaction (RT-qPCR) were designed using NCBI Primer-BLAST (Table 1). In addition, *β-actin* (XM_060405599.1) was used as the endogenous control.

Gene expression levels were quantified by RT-qPCR using PowerUp™ SYBR™ Green Master Mix (Thermo Fisher Scientific, Waltham, MA, USA; REF: A25742) and specific primers in a 20 μL reaction volume. RT-qPCR was performed under the following cycling conditions: initial denaturation at 95 °C for 2 min, followed by 40 cycles of denaturation at 95 °C for 15 s, combined annealing/extension at 60 °C for 1 min. Three biological replicates were analyzed for each tissue, and each sample was measured in triplicate. Quantification cycle (Cq/Ct) values were determined using the automatic threshold setting of QuantStudio Design and Analysis Software v 2.8 (Applied Biosystems, Thermo Fisher Scientific, Waltham, MA, USA). Relative expression levels of the target genes were calculated using the 2^^−ΔΔCt^ method.

### 2.3. Genomic DNA Isolation and SNP Genotyping

Genomic DNA was extracted using a GeneJET Genomic DNA Purification Kit (Thermo Fisher Scientific, USA; REF: K0722). The DNA samples were quantified using a Nanodrop^TM^ 1000 spectrophotometer (Thermo Scientific, Waltham, MA, USA), and all DNA samples were diluted to 50 ng/μL and stored at −40 °C.

The primer sequences for the ovine *DGAT1* locus were retrieved from the study by Yang et al. (2011) [20]. The primers for the ovine *CAST* variation were sourced from the research conducted by Gorlov et al. (2016) [21], while the primers for the SNPs (C114G and G116A) located in exon 1 of *IGF1* were derived from Behzadi et al. (2015) [22]. For the previously reported variations (C355T site (C/T), C71G site (C/G), and C330G site (C/G)) in the sheep *POU1F1* gene (Bai et al. 2016) [23], we designed new primers according to the reference sequence (NC_056054.1) using NCBI Primer-BLAST (https://www.ncbi.nlm.nih.gov/tools/primer-blast/). The primers were synthesized by the National Center for Biotechnology (Astana, Kazakhstan).

A total reaction volume of 20 μL (10 μL Master Mix, 0.3 μL of each primer, 0.8 μL of DNA and 8.6 μL of ddH2O) was used for the PCR amplification. PCR amplification was performed under the following cycling conditions: an initial denaturation at 95 °C for 3 min, followed by 35 cycles of denaturation at 95 °C for 30 s, annealing at 63 °C for 30 s, and extension at 72 °C for 1 min. A final extension was carried out at 72 °C for 5 min. The PCR products were visualized on a 3.5% agarose gel and sequenced at the laboratory of the Agricultural Biotechnology Research Center of Saken Seifullin Kazakh Agrotechnical University (Astana, Kazakhstan). All PCR amplicons were validated by Sanger sequencing using the forward primer. The obtained chromatograms were analyzed using Chromas v2.6.6 (Technelysium Pty Ltd., South Brisbane, QL, Australia). Base calls were accepted only when clear, well-defined, non-overlapping peaks were observed. Heterozygous genotypes were identified by the presence of two distinct peaks at the same nucleotide position. Samples with ambiguous or low-quality chromatograms were re-examined, and sequencing was repeated when necessary to confirm the genotype.

### 2.4. Statistical Analyses

Statistical analysis of gene expression data was performed using GraphPad Prism version 10.0 (GraphPad Software, San Diego, CA, USA).

Population genetic parameters, including genotypic and allelic frequencies, were calculated using Nei’s method [24]. The chi-square test was utilized to assess the Hardy–Weinberg equilibrium (HWE). Associations between *DGAT1* genotypes and growth traits were analyzed using one-way analysis of variance (ANOVA) in SPSS (version 25.0, IBM Corporation, Armonk, NY, USA) according to the following linear model:

*Y_ij_* = *μ* + *G_i_* + *e_ij_* (1)

where *Y_ij_* is the observed phenotypic value of the trait, *μ* is the overall mean, *G_i_* is the fixed effect of genotype, and *e_ij_* is the random residual error. No covariates were included in the model because all animals were adult females of similar age and were managed under similar conditions. When a significant genotype effect was detected, pairwise comparisons among genotype means were performed using Tukey’s honestly significant difference (HSD) test. A *p* value of less than 0.05 was deemed statistically significant.

## 3. Results

### 3.1. Relative mRNA Expression of the Candidate Genes

**Figure 1:**
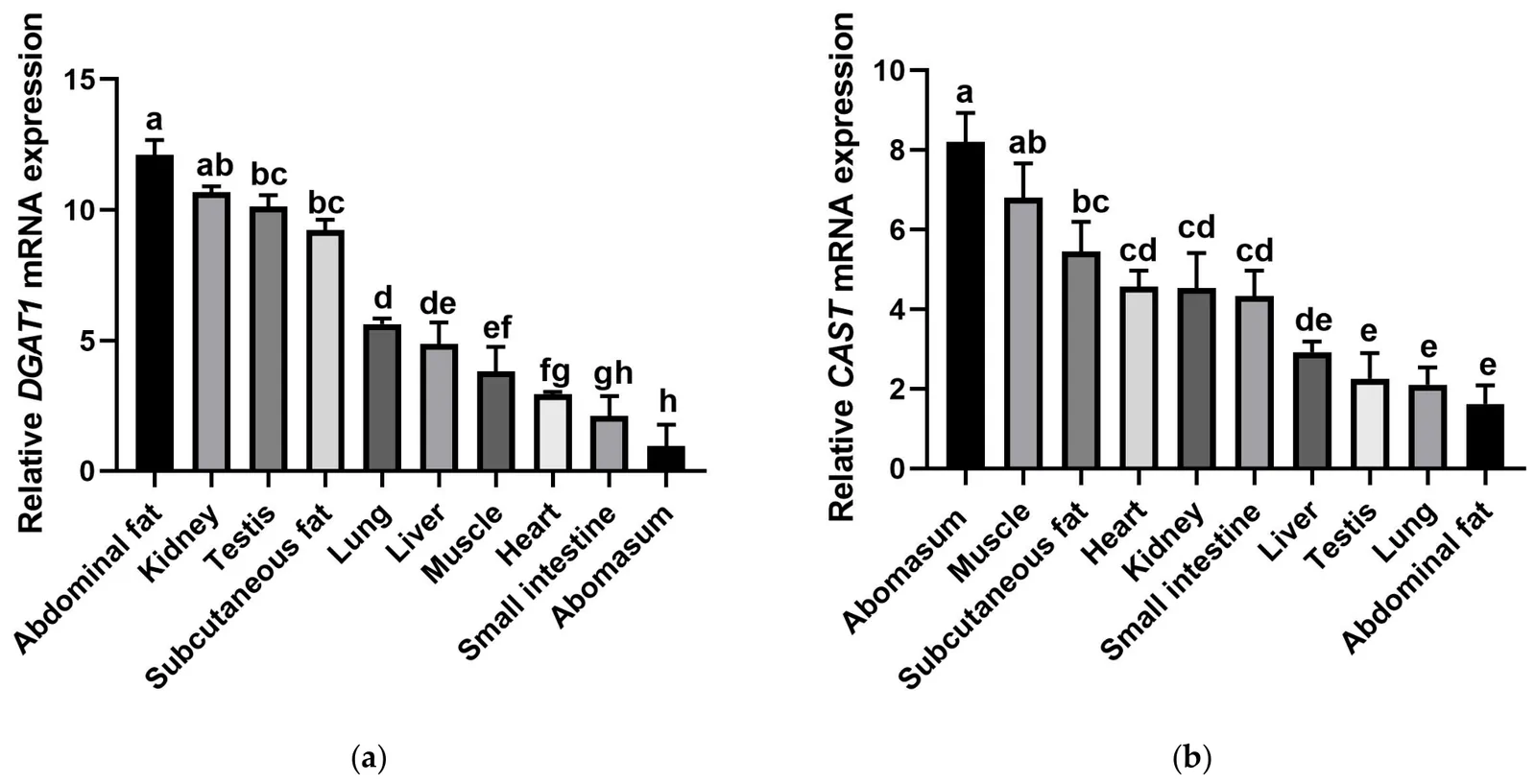

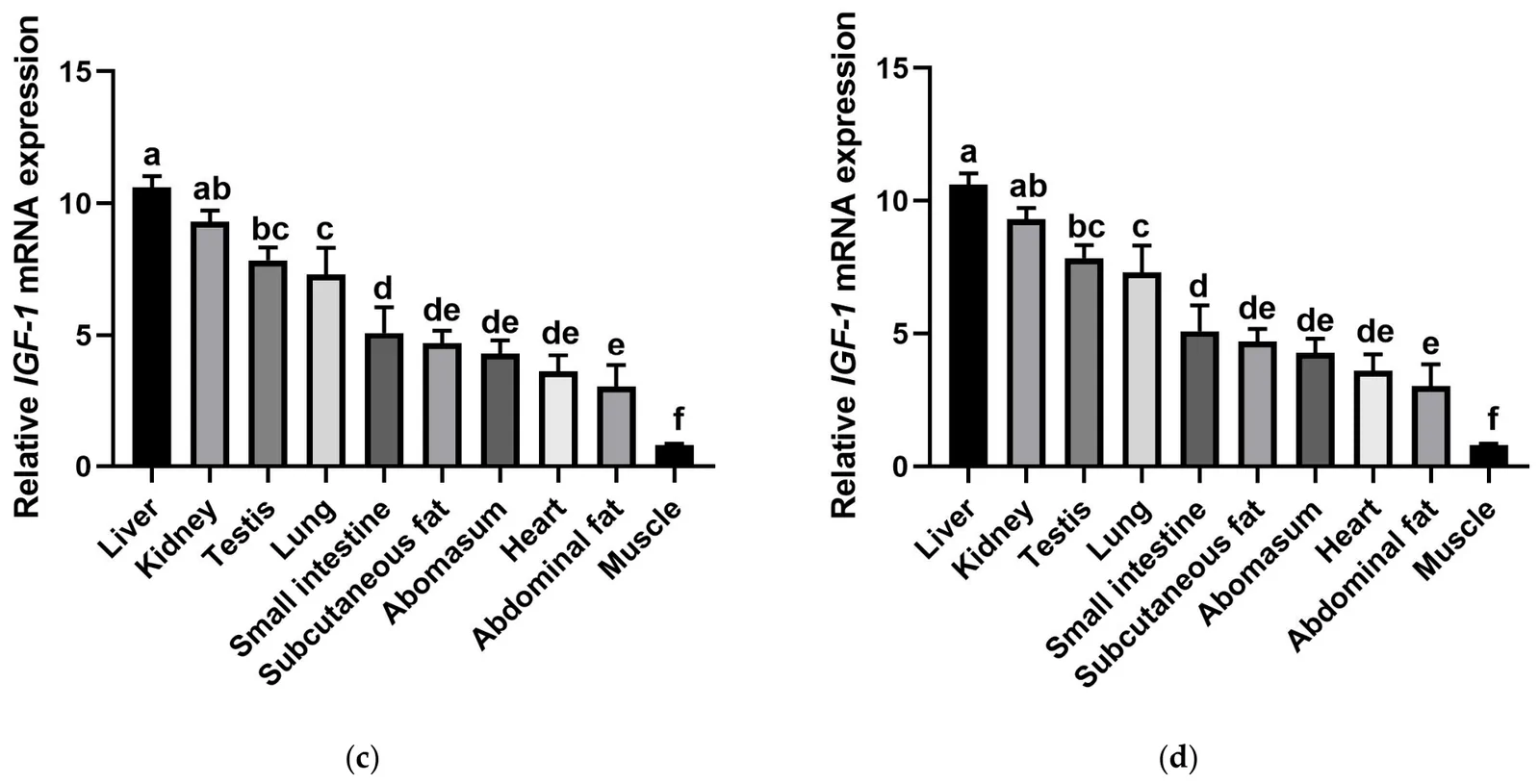
Relative mRNA expression levels of the candidate genes in sheep. (**a**) Relative mRNA expression levels of the *DGAT1* gene. (**b**) Relative mRNA expression levels of the *CAST* gene. (**c**) Relative mRNA expression levels of the *IGF-1* gene. (**d**) Relative mRNA expression levels of the *POU1F1* gene. Data are presented as mean ± SD from three biological replicates (*n* = 3), with each biological replicate analyzed in technical triplicate. Different lowercase letters indicate significant differences among tissues (*p* < 0.05).

Relative *DGAT1* mRNA expression levels varied significantly among different tissues, with the highest expression observed in abdominal fat, although it was not significantly different from that in the kidney (*p* > 0.05). Significant differences were detected among most tissues (*p* < 0.05), whereas several tissues exhibited comparable expression levels (*p* > 0.05), as indicated in Figure 1.

Similarly, relative *CAST* mRNA expression levels differed significantly among the examined tissues (*p* < 0.05). The highest mean expression was observed in the abomasum (Figure 1). However, its expression was not significantly different from that in the muscle (*p* > 0.05). Likewise, no significant differences were detected between several other tissue pairs, such as the liver, kidney, the heart, and small intestine (*p* > 0.05).

*IGF-1* mRNA expression exhibited significant tissue-specific differences (*p* < 0.05). The highest expression was observed in the liver, although it was not significantly different from that in the kidney (*p* > 0.05). Similarly, the kidney, testis, and lung, as well as the small intestine, subcutaneous fat, abomasum, heart, and abdominal fat, exhibited comparable expression.

*POU1F1* expression was highest in the testis, but it did not differ significantly from that in the abomasum, heart, kidney, or muscle (*p* > 0.05). However, it was significantly higher than that in the small intestine, abdominal fat, liver, lung, and subcutaneous fat (*p* < 0.05). Expression levels in the abomasum, heart, kidney, and muscle were comparable to those in the small intestine, abdominal fat, and liver, but were significantly higher than those in the lung and subcutaneous fat. No significant differences were observed among the small intestine, abdominal fat, liver, and lung (*p* > 0.05), whereas subcutaneous fat showed the lowest expression.

### 3.2. Identification of Polymorphisms and Genetic Parameters

**Figure 2:**
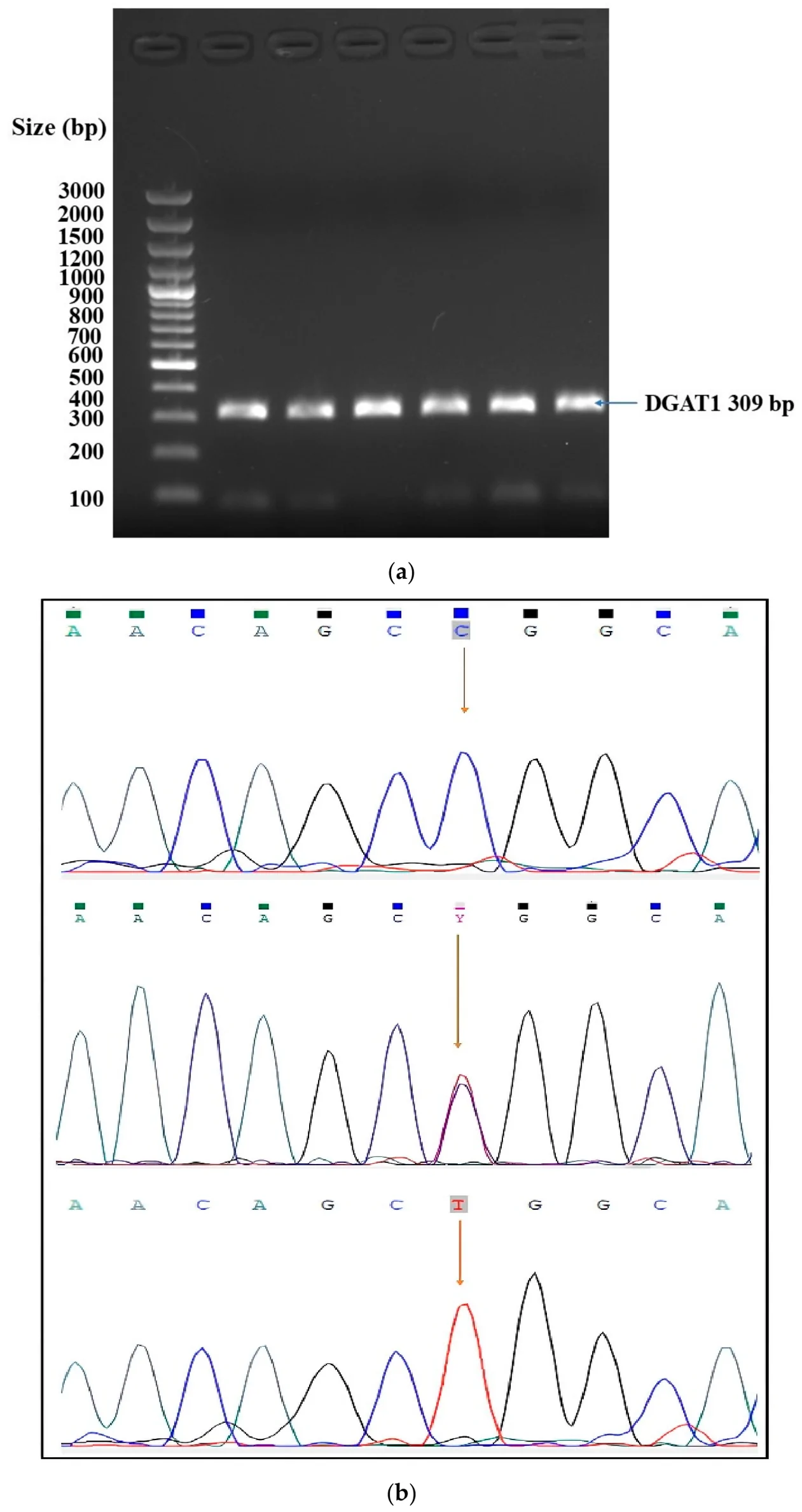
(**a**) The gel electrophoresis and (**b**) sequencing map of the DGAT1-SNP site.

After testing the *DGAT1*, *CAST*, *IGF1*, and *POU1F1* primers in Degeres samples, the PCR and sequencing results identified a previously described synonymous SNP (c.1461 C > T; GCC(Ala)→GCT(Ala)) within exon 17 of the *DGAT1* gene in the Degeres population (Figure 2). This SNP exhibited three genotypes, including the homozygous “CC”, heterozygous “CT”, and homozygous “TT” (Table 2). At the *DGAT1* site, the “CC” genotype was the most prevalent compared with the “TT” and “TC” genotypes (Table 2). Likewise, the “C” allele frequency (0.950) was markedly higher than that of the “T” allele (0.050). Moreover, this locus deviated from HWE (*p* < 0.05). The polymorphism information content (PIC) value indicated that the detected SNP in the Degeres population was characterized by low polymorphism (0 < PIC ≤ 0.25) (Table 2).

### 3.3. Association of SNP with Growth Traits in Degeres Sheep

The association analysis between the SNP and growth traits is presented in Table 3. A statistically significant association was detected for chest width (*p* < 0.05) and individuals with the “TT” genotype exhibited a higher chest width compared to those with “TC” and “CC” genotypes. The latter two genotypes did not differ significantly from each other. However, no significant differences among genotypes were observed for other traits, including body weight, height at the withers, chest circumference, diagonal body length, chest depth, and cannon circumference (*p* > 0.05).

These results suggest that the SNP may influence chest width, whereas it does not appear to influence the other measured growth traits in the studied population.

## 4. Discussion

Molecular-assisted selection can significantly accelerate the rate of genetic improvement in growth traits compared to traditional selection methods [25]. This is particularly important in sheep breeding, where generation intervals can be long, and rapid improvements can lead to better productivity and profitability. Moreover, it is essential when introducing new genetic material through crossbreeding. By studying variations in candidate genes, it is possible to better understand how specific genetic differences contribute to phenotypic traits, including production traits such as growth or carcass traits. To our knowledge, this is the first study to investigate the *DGAT1* polymorphism gene in the Degeres sheep population.

In the present study, one previously reported synonymous SNP in the *DGAT1* gene was identified, whereas no polymorphisms were detected at the other loci. In addition, the observed deviation from Hardy–Weinberg equilibrium may be attributed to several factors, including selection, population structure, inbreeding, genetic drift, or genotyping error. As the Degeres sheep represent a managed breeding population, non-random mating and selective breeding practices may have contributed to the observed genotype distribution. Therefore, further validation in larger independent populations is required to confirm the observed association. The relative mRNA expression analysis of *DGAT1* supported its key role in lipid metabolism and fat deposition. Consistent with our findings, Yang et al. (2011) also observed that the “CC” genotype was the most prevalent, and the “C” allele was the dominant allele across four Chinese sheep breeds [20]. Although the detected SNP showed low polymorphism, a significant association with chest width was observed in individuals with the “TT” genotype. Previously, it was reported that the “TT” genotype of this silent mutation was significantly associated with increased muscle marbling score, higher intramuscular fat content, and lower shear force and drip loss rates [26]. Despite being a synonymous variant that does not alter the amino acid sequence of *DGAT1*, synonymous variants may still influence gene function by affecting mRNA stability, translation efficiency, or by being in linkage disequilibrium with nearby functional variants [27]. For instance, a recent study by Zhen et al. (2026) demonstrated that this SNP in the *DGAT1* gene altered mRNA secondary structure and stability, thereby affecting *DGAT1* expression, triglyceride synthesis, and milk fat percentage in dairy sheep [28]. Since DGAT1 is an enzyme primarily involved in lipid metabolism, genetic variation in this gene may influence energy utilization and tissue development during growth, thereby contributing to differences in body conformation traits such as chest width. The primary function of DGAT1 is to catalyze the final step in the synthesis of triglycerides (TAGs) from diacylglycerol (DAG) and acyl-CoA [5]. Consequently, alterations in *DGAT1* function or regulation may affect fat deposition and body development, potentially contributing to the phenotypic differences observed in the present study. Abnormalities in *DGAT1* activity have also been linked to metabolic disorders, including obesity and insulin resistance [29].

Although no polymorphic SNP was detected in the *CAST* gene in the present study, the high expression of *CAST* in the abomasum and muscle suggests its role in muscle development and metabolism. Given the role of calpastatin in regulating calpain-mediated muscle protein degradation, variation in *CAST* expression or activity may influence muscle growth and tissue remodeling by altering the balance between protein synthesis and degradation [30,31]. Previously, Jawasreh et al. (2019) reported that CAST-HhaI was associated with body weight and longissimus muscle width in Awassi sheep [32]. Moreover, SNPs in the *CAST* gene were related to fatty acid compositions of longissimus thoracis muscle Sonid sheep [33]. Together, these findings suggest that *CAST* may contribute to phenotypic variation in growth and carcass traits through its role in regulating muscle protein turnover, providing biological support for the expression pattern observed in the present study.

In our study, the *IGF-1* gene was predominantly expressed in the liver, consistent with the liver being the primary site of IGF-1 synthesis in mammals and supporting its central role in growth and metabolic regulation [34]. Previously, SNPs in *IGF-1* were associated with birth weight, weaning weight, body weight, and other growth traits in sheep [35]. More recently, the g.857G>A SNP in this gene was found to influence the body weight in Indian sheep [36]. As IGF-1 is produced mainly in the liver in response to growth hormone (GH) stimulation, its high hepatic expression observed in the present study may contribute to the regulation of systemic growth and tissue development by promoting cell proliferation, differentiation, and metabolism [37]. These previous association studies, together with our expression results, further support the important role of IGF-1 in regulating growth-related traits in sheep.

The relative mRNA expression levels of the *POU1F1* gene showed that it displayed the most pronounced expression in the testes. Although POU1F1 is traditionally recognized as a pituitary transcription factor involved in growth hormone regulation, the *POU1F1* may also be associated with male reproductive traits [38]. It should be noted that the pituitary gland was not included among the tissues analyzed in the present study. Therefore, the relatively high *POU1F1* expression observed in testicular tissue in the present study may suggest an additional reproductive-related function beyond its canonical pituitary role. In addition, breed-specific genetic backgrounds, regulatory mechanisms, and physiological differences may also contribute to variation in *POU1F1* expression patterns. Further functional studies across diverse breeds and larger populations are needed to clarify the biological significance of this observation. POU1F1 regulates the expression of genes involved in the production of GH, which in turn stimulates the hepatic production of IGF-1. This creates a feedback loop where POU1F1 influences IGF-1 levels through its role in GH synthesis [39]. Its activity is vital for the differentiation and function of pituitary cells and particularly somatotrophs [40]. Genotypes of *POU1F1* were related to weaning weight in Iranian indigenous sheep breeds [17]. Also, *POU1F1* polymorphisms were correlated with growth traits in goats [41], cattle [42], and chickens [43].

A limitation of this study is that tissue expression analysis was performed using only three animals. Although the results provide preliminary insights into the expression patterns of the investigated genes, the limited sample size may have affected the statistical power and restrict the broader interpretation of the findings. Future studies involving larger sample sizes are needed to further validate and extend these observations. The animals included in the association analysis and tissue expression analysis differed in their demographic characteristics, which may contribute to variation in gene expression patterns and phenotypic traits. Therefore, the expression results should be interpreted as preliminary, and future studies using animals with matched sex and age backgrounds are recommended to better characterize the relationship between gene expression and phenotypic variation. Furthermore, the observed association between the *DGAT1* genotype and chest depth provides valuable insight into the potential relationship between genetic variation and body measurements. However, the relatively low frequency of the TT genotype may have affected the precision of the observed association. In addition, body weight and overall body frame may also contribute to variation in chest depth, future studies using multivariable statistical models could further clarify the independent effect of the *DGAT1* genotype.

## 5. Conclusions

In conclusion, the mRNA expression profiles of *DGAT1*, *CAST*, *IGF1*, and *POU1F1* showed clear tissue-specific patterns, indicating their coordinated roles in regulating growth, metabolism, and tissue development in Degeres sheep. One previously reported SNP (c.1461 C > T) was detected in the *DGAT1* gene in Degeres population. This SNP was found to be associated with chest width and sheep carrying “TT” genotype exhibited higher chest width. These findings suggest that this SNP may serve as a potential candidate marker for improving chest width in Degeres sheep. However, further studies involving larger datasets are needed to validate these associations and confirm their applicability in broader populations.

## Figures and Tables

**Table 1:** Primer sequence information for genes in sheep.

Genes	Primer Sequences	Size	Target Region	Application
*DGAT1*	F: CGCGCCAACAGACTACAAAG	126 bp	-	RT-qPCR
	R: AGGTGCCACTCTACGTACCA			
*CAST*	F: AGAGCCTCGTGTAGGTAGCA	99 bp	-	
	R: AAGAGGACTGAACCTTCCGC			
*IGF-1*	F: CAGTCACATCCTCCTCGCAT	112 bp	-	
	R: AGAGCATCCACCAACTCAGC			
*POU1F1*	F: CGTGATGTCCACAGCAACAG	95 bp	-	
	R: TTAAGCTCCCTGCCATCACG			
*β-actin*	F: ATCCTGCGGCATTCACGAAA	191 bp	-	
	R: GCTGGGCGCTCACCTTGAT			
***DGAT1***	**F: GCATGTTCCGCCTCTGG**	**309 bp**	**Exons 16–17**	PCR/Sanger sequencing
	**R: GGAGTCCAACACCCCTGA**			
*CAST*	F: TGGGGCCCAATGACGCCATCGATG	622 bp	Intron and exon	
	R: GGTGGAGCAGCACTTCTGATCACC			
*IGF1*	F: ATTACAAAGCTGCCTGCCCCTT	265 bp	Exon 1	
	R: CACATCTGCTAATACACCTTACCCG			
*POU1F1*	F: AGAAGTGGTGAGGGTTTGGT	499 bp	Exon and 3′ UTR	
	R: TGTGACAGAACTGGCCAAAA			

Note: Bold means polymorphism.

**Table 2:** Population genetic indices of DGAT1-SNP in the Degeres breed.

Locus	Frequencies					*Ho*	*He*	*Ne*	HWE *p*-Value	PIC
	Genotypes			Alleles						
DGAT1- SNP	TT (0.026) *n* = 10	TC (0.047) *n* = 18	CC (0.926) *n* = 352	T (0.050)	C (0.950)	0.047	0.095	1.105	0.001	0.091

Note: *Ho*, observed heterozygosity; *He*, expected heterozygosity; *Ne*, effective allele numbers; PIC, polymorphism information content; HWE, Hardy–Weinberg equilibrium.

**Table 3:** The association analysis between the DGAT1-SNP genotypes and growth traits in the Degeres breed.

Growth Traits	Observed Genotypes (Mean ± SD)			*p* Values
	TT (*n* = 10)	TC (*n* = 18)	CC (*n* = 352)	
BW, kg	49.410 ± 1.445	50.089 ± 1.433	49.940 ± 1.490	0.486
HW, cm	67.640 ± 0.961	67.606 ± 0.897	67.760 ± 0.975	0.754
ChC, cm	97.710 ± 1.139	97.572 ± 1.108	97.590 ± 1.064	0.938
DBL, cm	60.520 ± 0.925	60.506 ± 1.124	60.723 ± 1.009	0.565
ChD, cm	30.610 ± 1.302	30.506 ± 0.802	30.537 ± 0.988	0.964
ChW, cm	**^a^** **24.280 ± 3.305**	**^b^** **23.211 ± 0.680**	**^b^** **23.096 ± 0.800**	**0.001**
CC, cm	7.950 ± 0.255	7.867 ± 0.272	7.853 ± 0.291	0.572

Note: Cells with different letters, ^a,b^ differed significantly (*p* < 0.05). BW: body weight, HW: height at the withers, ChC: chest circumference, DBL: diagonal body length, ChD: chest depth, ChW: chest width, CC: cannon circumference.

## Data Availability

The dataset analyzed during the current study are available from the corresponding authors upon reasonable request.

## Abbreviations

The following abbreviations are used in this manuscript:

MAS	Marker-assisted selection
SNP	Single-nucleotide polymorphisms
DGAT1	Diacylglycerol O-acyltransferase 1
CAST	Calpastatin
IGF1	Insulin-like growth factor 1
POU1F1	Pituitary-specific transcription factor 1
PCR	Polymerase chain reaction

## References

[1] Adylkanova S.R., Sadykulov T.S., Kim G.L., Koishibayev A.M., Dolgopolova S.Y. (2018). The biological growth and development of lambs Degeres fat-tailed sheep breed. EurAsian J. BioSci..

[2] Begenova A., Bissengaliyev R., Kulmagambetov T., Nurgulsim K., Bekenova A., Otepova G., Akhatayeva Z. (2025). Molecular markers associated with growth, meat, and carcass traits in sheep: A review. Anim. Biotechnol..

[3] Li T., Xing F., Zhang N., Chen J., Zhang Y., Yang H., Peng S., Ma R., Liu Q., Gan S. (2024). Genome-wide association analysis of growth traits in Hu sheep. Genes.

[4] Araujo A.C., Carneiro P.L.S., Oliveira H.R., Lewis R.M., Brito L.F. (2023). SNP- and haplotype-based single-step genomic predictions for body weight, wool, and reproductive traits in North American Rambouillet sheep. J. Anim. Breed. Genet..

[5] Cheng X., Geng F., Pan M., Wu X., Zhong Y., Wang C., Tian Z., Cheng C., Zhang R., Puduvalli V. (2020). Targeting DGAT1 ameliorates glioblastoma by increasing fat catabolism and oxidative stress. Cell Metab..

[6] Bayraktar M., Shoshin O. (2022). Estimation of the associations between GH and DGAT1 genes and growth traits by using decision tree in Awassi sheep. Anim. Biotechnol..

[7] Mohammadi H., Shahrebabak M.M., Sadeghi M. (2013). Association between single nucleotide polymorphism in the ovine DGAT1 gene and carcass traits in two Iranian sheep breeds. Anim. Biotechnol..

[8] Khan M.Z., Ma Y., Ma J., Xiao J., Liu Y., Liu S., Khan A., Khan I.M., Cao Z. (2021). Association of DGAT1 with cattle, buffalo, goat, and sheep milk and meat production traits. Front. Vet. Sci..

[9] Tait R.G., Shackelford S.D., Wheeler T.L., King D.A., Keele J.W., Casas E., Smith T.P., Bennett G.L. (2014). CAPN1, CAST, and DGAT1 genetic effects on preweaning performance, carcass quality traits, and residual variance of tenderness in a beef cattle population selected for haplotype and allele equalization. J. Anim. Sci..

[10] Bhat Z., Morton J.D., Mason S.L., Bekhit A.E.-D.A. (2018). Role of calpain system in meat tenderness: A review. Food Sci. Hum. Wellness.

[11] Choi E., Duan C., Bai X.C. (2025). Regulation and function of insulin and insulin-like growth factor receptor signalling. Nat. Rev. Mol. Cell Biol..

[12] Meira A.N., Montenegro H., Coutinho L.L., Mourão G.B., Azevedo H.C., Muniz E.N., Machado A.L., Sousa-Jr L.P., Pedrosa V.B., Pinto L.F.B. (2019). Single nucleotide polymorphisms in the growth hormone and IGF type-1 (IGF1) genes associated with carcass traits in Santa Ines sheep. Animal.

[13] Kader Esen V., Esen S. (2023). Association of the IGF1 5′UTR polymorphism in meat-type sheep breeds considering growth, body size, slaughter, and meat quality traits in Turkey. Vet. Sci..

[14] Arikawa L.M., Mota L.F.M., Schmidt P.I., Frezarim G.B., Fonseca L.F.S., Magalhães A.F.B., Silva D.A., Carvalheiro R., Chardulo L.A.L., Albuquerque L.G. (2024). Genome-wide scans identify biological and metabolic pathways regulating carcass and meat quality traits in beef cattle. Meat Sci..

[15] El-Attrouny M.M., Iraqi M.M., Sabike I.I., Abdelatty A.M., Moustafa M.M., Badr O.A. (2021). Comparative evaluation of growth performance, carcass characteristics and timed series gene expression profile of GH and IGF-1 in two Egyptian indigenous chicken breeds versus Rhode Island Red. J. Anim. Breed. Genet..

[16] Bando H., Brinkmeier M.L., Castinetti F., Fang Q., Lee M.S., Saveanu A., Albarel F., Dupuis C., Brue T., Camper S.A. (2023). Heterozygous variants in SIX3 and POU1F1 cause pituitary hormone deficiency in mouse and man. Hum. Mol. Genet..

[17] Sadeghi M., Jalil-Sarghale A., Moradi-Shahrbabak M. (2014). Associations of POU1F1 gene polymorphisms and protein structure changes with growth traits and blood metabolites in two Iranian sheep breeds. J. Genet..

[18] Zhang Y., Cui W., Yang H., Wang M., Yan H., Zhu H., Liu J., Qu L., Lan X., Pan C. (2019). A novel missense mutation (L280V) within POU1F1 gene strongly affects litter size and growth traits in goat. Theriogenology.

[19] Ncube K.T., Dzomba E.F., Hadebe K., Soma P., Frylinck L., Muchadeyi F.C. (2022). Carcass quality profiles and associated genomic regions of South African goat populations investigated using goat SNP50K genotypes. Animals.

[20] Yang J., Zang R., Liu W., Xu H., Bai J., Lu J., Wu J. (2011). Polymorphism of a mutation of DGAT1 gene in four Chinese Indigenous sheep breeds. Asian J. Anim. Vet. Adv..

[21] Gorlov I.F., Shirokova N.V., Randelin A.V., Voronkova V.N., Mosolova N.I., Zlobina E.Y., Kolosov Y.A., Bakoev N.F., Leonova M.A., Bakoev S.Y. (2016). CAST/MspI gene polymorphism and its impact on growth traits of Soviet Merinoand Salsk sheep breeds in the South European part of Russia. Turk. J. Vet. Anim. Sci..

[22] Behzadi S., Miraei-Ashtiani S.R., Sadeghi M., Zamani P., Abdoli R. (2015). Association of IGF-1 gene polymorphisms with carcass traits in Iranian Mehraban sheep using SSCP analysis. Iran. J. Appl. Anim. Sci..

[23] Bai J.Y., Wang X., Yang Y.B., Zhang X.H., Pang Y.Z., Li H.W. (2016). Study on the polymorphism of POU1F1 gene in sheep. Rev. Bras. Zootec..

[24] Kazhgaliyev N., Nurgulsim K., Makhanbetova A., Ibrayev D., Shaikenova K., Hasen Z., Amantay S., Zhumagaziyeva S., Mukhametzharova I., Bakytzhan A. (2025). Genetic variation and mRNA expression of the ELOVL6 and CRTC2 genes in Kalmyk cattle. Anim. Biotechnol..

[25] Zhu L., Akhmet N., Bo D., Pan C., Wu J., Lan X. (2024). Genetic variant of the sheep E2F8 gene and its associations with litter size. Anim. Biotechnol..

[26] Xu Q.L., Chen Y.L., Ma R.X., Xue P. (2009). Polymorphism of DGAT1 associated with intramuscular fat-mediated tenderness in sheep. J. Sci. Food Agric..

[27] Zhang J., Qian W. (2025). Functional synonymous mutations and their evolutionary consequences. Nat. Rev. Genet..

[28] Zhen H., Zhou H., Wang Z., Li M., Hao Z., Che L., Ren C., Wang J., Wu X., Liu Y. (2026). A Synonymous SNP in DGAT1 Affects milk fat percentage of dairy sheep by regulating the stability of the mRNA to Change the viability, proliferation, and triglyceride levels of ovine mammary epithelial cells. FASEB J..

[29] Tsuda N., Kumadaki S., Higashi C., Ozawa M., Shinozaki M., Kato Y., Hoshida K., Kikuchi S., Nakano Y., Ogawa Y. (2014). Intestine-targeted DGAT1 inhibition improves obesity and insulin resistance without skin aberrations in mice. PLoS ONE.

[30] Sato K., Minegishi S., Takano J., Plattner F., Saito T., Asada A., Kawahara H., Iwata N., Saido T.C., Hisanaga S. (2011). Calpastatin, an endogenous calpain-inhibitor protein, regulates the cleavage of the Cdk5 activator p35 to p25. J. Neurochem..

[31] Schroder E.A., Wang L., Wen Y., Callahan L.A.P., Supinski G.S. (2021). Skeletal muscle-specific calpastatin overexpression mitigates muscle weakness in aging and extends life span. J. Appl. Physiol..

[32] Jawasreh K.I., Al-Amareen A.H., Aad P.Y. (2019). Relationships between Hha1 Calpastatin gene polymorphism, growth performance, and meat characteristics of Awassi sheep. Animals.

[33] Guo X., Li T., Lu D., Yamada T., Li X., Bao S., Liu J., Borjigin G., Cang M., Tong B. (2023). Effects of the Expressions and variants of the CAST gene on the fatty acid composition of the longissimus thoracis muscle of grazing Sonid sheep. Animals.

[34] Adamek A., Kasprzak A. (2018). Insulin-like growth factor (IGF) system in liver diseases. Int. J. Mol. Sci..

[35] Sako T., Tyasi T.L., Ng’ambi J. (2024). Insulin-like growth factor 1 gene and growth traits correlations in sheep: A systematic review. Am. J. Anim. Vet. Sci..

[36] Kumar S., Dahiya S.P., Magotra A., Ratwan P., Bangar Y. (2023). Influence of single nucleotide polymorphism in the IGF-1 gene on performance and conformation traits in Munjal sheep. Zygote.

[37] Kineman R.D., Del Rio-Moreno M., Waxman D.J. (2025). Liver-specific actions of GH and IGF1 that protect against MASLD. Nat. Rev. Endocrinol..

[38] Li G., Peñagaricano F., Weigel K.A., Zhang Y., Rosa G., Khatib H. (2012). Comparative genomics between fly, mouse, and cattle identifies genes associated with sire conception rate. J. Dairy Sci..

[39] Romero C.J., Pine-Twaddell E., Sima D.I., Miller R.S., He L., Wondisford F., Radovick S. (2012). Insulin-like growth factor 1 mediates negative feedback to somatotroph GH expression via POU1F1/CREB binding protein interactions. Mol. Cell. Biol..

[40] Wallis M. (2018). Evolution of the POU1F1 transcription factor in mammals: Rapid change of the alternatively-spliced β-domain. Gen. Comp. Endocrinol..

[41] Zhu H., Zhang Y., Bai Y., Yang H., Yan H., Liu J., Shi L., Song X., Li L., Dong S. (2019). Relationship between SNPs of POU1F1 gene and litter size and growth traits in Shaanbei White cashmere goats. Animals.

[42] Zhang C., Liu B., Chen H., Lan X., Lei C., Zhang Z., Zhang R. (2009). Associations of a HinfI PCR-RFLP of POU1F1 gene with growth traits in Qinchuan cattle. Anim. Biotechnol..

[43] Manjula P., Choi N., Seo D., Lee J.H. (2018). POU class 1 homeobox 1 gene polymorphisms associated with growth traits in Korean native chicken. Asian-Australas. J. Anim. Sci..

